# Zika virus infection reduces hemocyte numbers and induces hemocyte aggregation in adult *Drosophila melanogaster*

**DOI:** 10.17912/micropub.biology.002148

**Published:** 2026-08-04

**Authors:** Sneh Harsh, Sreeradha Mallick, George Joseph Chakkalakkal, Ioannis Eleftherianos

**Affiliations:** 1 George Washington University, Washington, DC, United States; 2 New York University School of Medicine; 3 Queen's University Belfast, Belfast, NIR, United Kingdom

## Abstract

Despite recent advances in the prevalence of Zika virus (ZIKV), our understanding of the host cellular components of the innate immune system that suppress ZIKV infection remains incomplete. Here, we have examined the impact of ZIKV on the hemocytes of *Drosophila melanogaster* . We describe for the first time that the introduction of ZIKV particles into the hemolymph of wild-type adult flies leads to two substantial effects: decrease in hemocyte numbers and increase in hemocyte aggregation. This is significant information which suggests the functional immune role of hemocytes in the host response to ZIKV and probably other flaviviruses.

**Figure 1. Zika virus infection affects cellular immune responses in Drosophila melanogaster adults:** (A) Numbers of circulating free (non-aggregated) hemocytes in the hemolymph of *D. melanogaster* adult flies injected with *Escherichia coli* (Ec) (positive control), phosphate-buffered saline (PBS) (negative control), *Drosophila C* virus (DCV), or Zika virus (ZIKV); (B) Quantification of hemocyte aggregation in the hemolymph of *D. melanogaster* adult flies injected with Ec, PBS, DCV or ZIKV; (C) Images of hemocyte aggregates in the hemolymph of *D. melanogaster* adult flies challenged with Ec, PBS, DCV or ZIKV. Each hemocyte count and aggregation assay involved three biological replicates consisting of 50 wild-type female flies per treatment and was repeated five independent times (**P < 0.001, *P < 0.05, ns = non-significant).

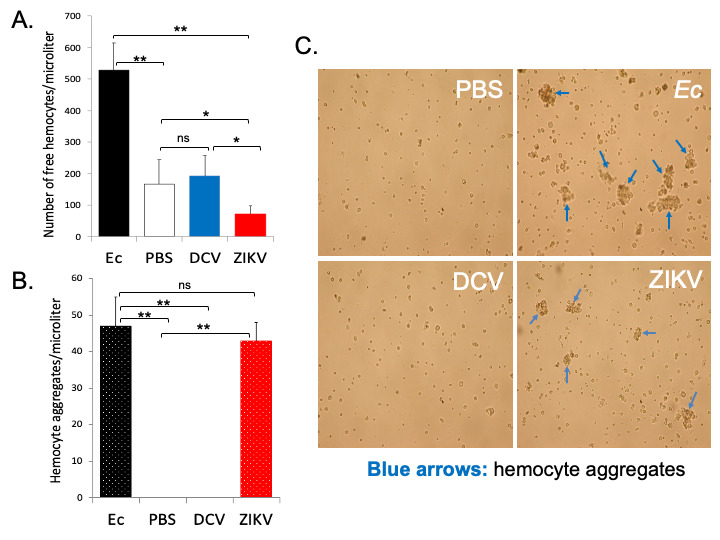



## Description

Zika Virus (ZIKV) is a vector-borne virus which has recently expanded its range dramatically and severe outbreaks appeared in several parts of the world. Because vector control is the only viable alternative for alleviating the disease, a thorough understanding of host-virus interaction is critical (Saiz, 2019). Thus, there is an urgent need to use a well-characterized genetic model for identifying and characterizing the number and types of host factors that participate in the response against ZIKV. Because host innate immunity is evolutionarily conserved across many phyla, investigating the effect of ZIKV infection on the innate immune responses of animal models is particularly informative because it can lead to the identification of anti-ZIKV mechanisms in vertebrates, including humans (Litman et al., 2005). Elucidating the cellular functions and their individual components regulating host anti-ZIKV defense will potentially lead to the development of innovative concepts and means for the efficient control of this disease.

The use of the fruit fly model host *D. melanogaster* has led to important advances in the characterization of the molecular processes leading to the activation of innate immune responses against pathogenic microbes causing disease, including viral pathogens. Apart from those viruses that naturally infect *Drosophila* , the fly is also an outstanding system for dissecting host interactions with human pathogenic viruses, understanding virus induced pathogenesis, and analyzing the in vivo function of viral genes encoding immune invasion or subversion factors, which can be further validated in studies involving mammalian models (Tafesh-Edwards and Eleftherianos, 2020). Analyzing the specific function of viral components forms an elegant strategy to promote our understanding of the molecular basis of viral diseases. Due to significant conservation between the fly and mosquito immune systems, such studies are crucial for elucidating the signaling pathways that operate in the mosquito antiviral innate immune response or identifying the cellular mechanisms that modulate the complex interactions between mosquito vectors and viral pathogens (King, 2020; Cardoso-Jaime et al., 2025).

Using the *D. melanogaster* model, previous work has shown that ZIKV infection regulates the immune, metabolic and neuronal responses. Injection of ZIKV particles into the hemolymph of adult wild-type flies induces the conserved signaling pathways of RNA interference (RNAi) and Janus Kinase and Signal Transducer and Activator of Transcription (Jak/Stat), and triggers the melanization response, which connects humoral and cellular immune reactions. Also, the contribution of the phenoloxidase system to the survival and defense of *D. melanogaster* against ZIKV is due to the activity of the prophenoloxidase genes *PPO1* and *PPO2* , which promote the survival and trigger melanin formation in the hemolymph and at the wound site on the cuticle of the injected flies. ZIKV has been further found to target the brain of adult flies, where it mainly replicates in the neurons. The *D. melanogaster* immune response in the brain involves the activation of the RNAi signaling as well as an inflammatory reaction which is regulated by the Imd pathway and leads to the induction of the *D. melanogaster* Stimulator of Interferon Genes (dSTING) homolog. Expression of dSTING in the neurons generates an autophagy response which restricts ZIKV replication and promotes fly survival. Silencing Ref(2)P (the fly ortholog of the mammalian polyubiquitin-binding scaffold protein p62) in the brain of adult *D. melanogaster* elevates ZIKV replication in fly heads. In addition, previous work in the fly model leads to the proliferation of intestinal stem cells in the midgut of wild type adults and alters insulin signaling and lipid metabolism in Dicer-2 mutants and advances the resistance to ZIKV in female flies carrying the *Wolbachia* endosymbionts (Eleftherianos and Mallick, 2025 and references therein).

The *D. melanogaster* immune system is traditionally divided into two tightly interconnected components: cellular responses (mediated by hemocytes, i.e. the insect blood cells) and humoral responses (mediated by soluble factors in the hemolymph). While they are usually studied separately, significant overlap exists because many humoral factors affect hemocyte functions, and hemocytes are a major source of humoral molecules (Yu et al. 2022). Cellular immune responses in flies mainly include the functions of phagocytosis (primarily performed by plasmatocytes, which are the most abundant hemocytes and function as professional phagocytes, acting similarly to vertebrate macrophages to engulf small microorganisms like bacteria), nodulation (when bacteria are too numerous for single-cell phagocytosis, hemocytes bind together with pathogens to form large clusters, which are later melanized), and encapsulation (used for large pathogens like parasitoid wasp eggs or nematodes which are too big to be phagocytosed, where lamellocytes wrap around the invader). Nodulation and encapsulation are frequently followed by the rapid melanization response, which aids wound healing and involves the activation of the phenoloxidase cascade that takes place after the rupture of crystal cells. *Drosophila* hemocyte aggregation refers to the clustering of blood cells (primarily plasmatocytes), creating a specialized sessile compartment in which hemocytes differentiate and group together to immobilize the pathogen (Gold and Brückner, 2015; Parsons and Foley, 2016). Recent single-cell RNA sequencing has identified even more diverse hemocyte sub-types, including primocytes and thanacytes, further highlighting the complexity of the fly blood system (Fu et al., 2020).

Previous works have uncovered a role for cellular immunity in the *Drosophila* antiviral response, suggesting a minor role for autophagy and major contribution of phagocytosis against certain viral pathogens (Tassetto et al., 2017). The goal of this study was to examine the effect of ZIKV infection on the total number of hemocytes and their aggregation response in *D. melanogaster* adult flies.

Because certain cellular mechanisms mediated by circulating macrophage-like immune cells contribute to virus-specific immune responses in the fly (Lamiable et al., 2016), we have analyzed *D. melanogaster* hemocyte-based immunity against ZIKV infection (Figure 1). We have quantified the number of circulating free (non-aggregated) hemocytes 24 hours after injection of *D. melanogaster* *w ^1118^* adult flies with either 11,000 PFU/fly of ZIKV strain MR766 or *Drosophila* C virus (DCV), which served as a viral control because previous studies have shown that hemocytes are not major contributors to the immune response against DCV infection (Lamiable et al., 2016). We have also injected flies with 11,000 CFU/fly of *E. coli* K12 (induces proliferation of hemocytes) or PBS (negative control) (Ghosh et al., 2018). We have found that hemocyte numbers in the hemolymph of ZIKV-infected flies are considerably lower compared to those in the DCV-infected individuals and compared to the control treatments ( Figure 1A ). To assess whether changes in hemocyte numbers influence their functional properties, we have visually estimated the ability of hemocytes to aggregate, which serves as a proxy for the cellular immune response to the presence of microbial intruders in *D. melanogaste* r (Sigle and Hillyer, 2016). Interestingly, we have observed the formation of hemocyte aggregates in the hemolymph of *E. coli* - and ZIKV-infected flies with no statistically significant difference between the two treatments ( Figure 1B and 1C). However, we have found no hemocyte aggregates in the hemolymph of DCV- and PBS-injected flies ( Figure 1B and 1C). These results imply that ZIKV infection can induce cellular immune responses mediated by hemocytes in *D. melanogaster* adult flies.

We consistently observed across independent experiments that hemocyte aggregates formed following *E. coli* infection appeared larger than those induced by ZIKV, although aggregate size was not quantified in the present study. One possible explanation is that bacterial pathogens, because of their substantially larger size and greater structural complexity than viral particles, require the recruitment of more hemocytes to form larger cellular aggregates capable of immobilizing and eliminating the invading microorganisms, consistent with the established role of hemocyte aggregation and nodulation in antibacterial immunity (Gold and Brückner, 2015; Parsons and Foley, 2016). In contrast, the smaller aggregates observed following ZIKV infection may reflect a distinct cellular response to viral infection or differences in the signals that regulate hemocyte recruitment and activation. Future studies that quantitatively measure aggregate size and composition and determine how these parameters relate to pathogen clearance will be necessary to test these possibilities.

Although the present study does not investigate the mechanism underlying the reduction in circulating free hemocytes following ZIKV infection, one possible explanation is that ZIKV may promote hemocyte apoptosis or other forms of cell loss. Alternatively, the decrease in circulating hemocytes may result from enhanced recruitment of hemocytes into sessile compartments or sites of immune activation, where they participate in aggregate formation (Gold and Brückner, 2015; Parsons and Foley, 2016). Interestingly, both ZIKV and *E. coli* infection induced hemocyte aggregation, but only *E. coli* increased the number of circulating free hemocytes, consistent with previous reports showing that bacterial infection stimulates hemocyte proliferation (Ghosh et al., 2018). In contrast, ZIKV infection reduced the number of circulating free hemocytes despite promoting aggregation, suggesting that viral infection may differentially regulate hemocyte homeostasis through mechanisms distinct from those activated during bacterial infection. Future studies examining hemocyte apoptosis, proliferation, and tissue localization during ZIKV infection will be important for distinguishing among these possibilities and defining the mechanisms responsible for the observed reduction in circulating hemocytes.

Future work will focus on revealing the identity of ZIKV molecular components responsible for the observed cellular immune effects. This could be done through the characterization of cellular immune activity (e.g., hemocyte-mediated phagocytosis, hemocyte aggregation and encapsulation) in *D. melanogaster* adult flies overexpressing ZIKV proteins using hemocyte-specific drivers (Neyen et al., 2014). Also, another aspect to investigate will be to identify the types of *D. melanogaster* hemocytes that participate in cellular immune responses to ZIKV and dissecting the interrelationship between fly humoral and cellular immune mechanisms directed against ZIKV infection.

An important consideration is that *D. melanogaster* has not co-evolved with ZIKV and is therefore not a natural host for this flavivirus, unlike *Aedes* mosquitoes, which serve as its primary vectors (King, 2020; Eleftherianos and Mallick, 2025). Consequently, some of the cellular immune responses observed in *D. melanogaster* may reflect interactions with a non-natural pathogen and could differ from those elicited by viruses that naturally infect *D. melanogaster* , such as DCV. Furthermore, although flies and mosquitoes possess evolutionarily conserved innate immune pathways and broadly similar hemocyte populations, important differences exist in their cellular immune systems (King, 2020; Cardoso-Jaime et al., 2025). For example, lamellocytes are present in flies but have not been identified in mosquitoes, indicating that comparisons of hemocyte responses between these insects should be interpreted with appropriate caution (Gold and Brückner, 2015; Cardoso-Jaime et al., 2025). Future comparative studies using mosquito models will be important for determining the extent to which the mechanisms identified in *D. melanogaster* are conserved in the natural vectors of ZIKV.

In conclusion, our results strikingly illustrate for the first time that introduction of ZIKV particles into the hemolymph of wild-type adult flies substantially decreases the number of circulating hemocytes and in contrast induces the hemocyte aggregation response. Previous work has shown that ZIKV infection modifies the NF-κB signaling and interacts with the phenoloxidase/melanization response in the fly (Harsh et al., 2018; Harsh et al., 2022; Tafesh-Edwards and Eleftherianos, 2023). The current findings build on the previous information indicating that ZIKV infection can regulate the cellular immune response in *D. melanogaster* . Altogether, this is significant knowledge because it reveals that ZIKV can shape many aspects of the host innate immune system to establish infection. Extending this research will be important for developing innovative means for the efficient control of this disease.

## Methods

**Fly and viral stocks.** *Drosophila melanogaster* adult flies and viral stocks were maintained and amplified using previously described protocols (Harsh et al., 2018; Croker et al., 2007). Adult female flies (5-7 days old) of the *D. melanogaster* *w ^1118^* line (stock 3605, Bloomington *Drosophila* Stock Center) were used in the hemocyte analysis experiments. Flies of the *w ^1118^* fly stock were free of *Wolbachia* endosymbionts. They were reared on Bloomington *Drosophila* Stock Center cornmeal food (LabExpress) supplemented with yeast (Carolina Biological Supply) and maintained in an incubator at 25°C and a 12-hour photoperiod. Viral stocks used in fly injections involved the ZIKV strain MR766 and *Drosophila* C virus (DCV), which was included as a viral control for comparison with ZIKV infection.

**Fly injections.** Adult fly infections with ZIKV and DCV were carried out through an intrathoracic nano-injection (Nanoject III apparatus, Drummond Scientific) of 100 nl of each viral suspension (approx. 11,000 PFU/fly). Injection of 100 nl of PBS (pH 7.5) served as the negative control. At 24 hours post injection, flies were anesthetized with carbon dioxide and hemolymph collection was performed using a previously established method involving the injection of incubation and collection solutions (Shokal and Eleftherianos, 2017).

**Hemocyte experiments.** For counting circulating free (non-aggregated) hemocytes, 10 μL of hemolymph samples were transferred to a hemocytometer and hemocyte numbers were estimated using 40× magnification of a compound microscope (Olympus CX21). Aggregated hemocytes were excluded from this analysis and were quantified separately in the hemocyte aggregation assay. For fly hemocyte aggregation assays, hemolymph samples were transferred to 96-well microtiter plates and hemocyte microaggregates (>5 cells) were counted using an inverted microscope (Novakova and Dolezal, 2011). Images were analyzed using Nikon Software Suite at 10X magnification. Each hemocyte number and hemocyte aggregation assay involved three biological replicates consisting of 50 flies per treatment and every assay was repeated five times.

**Statistical analysis.** GraphPad Prism 9 software was used for statistical analysis of the hemocyte numbers and hemocyte aggregates in the hemolymph of *D. melanogaster* adult flies challenged with *E. coli* , PBS, DCV or ZIKV. Statistics were performed on data obtained from five independent experiments.Comparisons of hemocyte numbers and aggregates between the different treatments were performedusing one-way analysis of variance (ANOVA) and Dunnet’s multiple comparisons tests in GraphPad Prism 9.All error bars represent standard error of mean.
